# Water quality and flow dataset from a degraded peatland catchment: Storm events and treatment pond performance

**DOI:** 10.1016/j.dib.2026.113070

**Published:** 2026-07-10

**Authors:** Lipe R.D. Mendes, Behzad Mozafari, Catharine M. Pschenyckyj, Michael Bruen, Fiachra O'Loughlin, Florence Renou-Wilson

**Affiliations:** a School of Biology and Environmental Science, University College Dublin, D04 N2E5, Dublin, Ireland; b School of Civil Engineering, University College Dublin, D04 V1W8, Dublin, Ireland; c School of Health and Life Sciences, Teesside University, TS1 3BX, Middlesbrough, United Kingdom

**Keywords:** Nutrients, Carbon, Ions, Peat extraction, Drainage, Effluents, Hydrochemistry, Water treatment

## Abstract

This dataset provides high-resolution hydrological and water quality data collected at the outlet of a 16.4 ha industrial cutaway peatland catchment in Ireland. The dataset captures 14 storm events of varying intensities between March 2021 and March 2023, with six events peaking below 15 L/s and eight exceeding 30 L/s. Water samples were collected using an automated ISCO 6712 sampler, paired with flow and temperature measurements recorded at high frequency. The dataset includes parameters such as total dissolved carbon (TDC), dissolved organic carbon (DOC), total dissolved nitrogen (TDN), nitrate (NO₃-N), total ammonia (NH₃-N), ions (Na, K, Ca, Mg, Cl, SO₄), pH, conductivity, and SUVA_254_ (a measure of carbon aromaticity). Flow data were recorded at 1–5-minute intervals using an ISCO 2150 area velocity flow module, capturing detailed hydrological responses to storm events. Water quality data were collected from a treatment pond receiving effluents from the catchment, allowing for an evaluation of pollutant retention and potential nature-based treatment performance. Grab sampling campaigns were conducted at the pond’s inlet and outlet from March–October 2021 and September 2022–April 2023, providing additional data on nutrient and ion dynamics. The pond had a median hydraulic retention time of 15 days, with a highly variable mean of 90 ± 551 days from July 2022 to June 2023. The dataset is structured in three Excel files—Storm Events, Flow, and Grab Sampling—and facilitates analyses of peatland drainage impacts on downstream water quality, storm-driven pulses in pollutant transport, and the effectiveness of treatment ponds in mitigating nutrient and carbon fluxes. By integrating high-frequency flow and water quality data, this dataset enables comprehensive evaluation of hydrological and biogeochemical responses in degraded peatland environments. It is valuable for hydrological modelling applications, runoff-driven nutrient transport studies, and evaluations of climate change effects on peatland effluent dynamics. Researchers can reuse this dataset for comparative studies on storm event hydrochemistry, treatment pond efficiencies, and peatland restoration impacts. The dataset is openly available, offering valuable insights into water quality management and the environmental effects of peatland drainage.

Specifications Table

**Table:** 

Subject	Earth & Environmental Sciences
Specific subject area	Water quality dynamics and treatment efficiency of an excavated pond at the edge of a degraded peatland catchment subject to peat extraction.
Type of data	Table; Raw, Analysed, Processed.
Data collection	Water quality and flow data were collected at the outlet of an industrial cutaway peatland in Ireland during 14 storm events (March 2021–March 2023) and from a treatment pond (March–October 2021, September 2022–April 2023). An ISCO 6712 sampler captured stormwater following heavy rainfalls based on water level thresholds, sampling more frequently during flow rise and peak and less often during flow recession. Flow was recorded with an ISCO 2150 area velocity flow module and ISCO 730 bubbler flow module. Grab samples were taken at the pond inlet and outlet. Parameters included pH, conductivity, nutrients, SUVA_254_, ions (Na, K, Ca, Mg, Cl, SO_4_), turbidity, and BOD₅. Flow was estimated via rating curves.
Data source location	The data was collected from the effluents of Corralanna, a raised bog used for peat extraction in Ireland (53°41′54.8"N 7°24′53.6"W). It is stored at University College Dublin, Ireland.
Data accessibility	Repository name: Mendeley Data Digital Object Identifier (DOI): 10.17632/4bt4jwjdwm.2 10.17632/rghjtw9p55.2 10.17632/wrt4dk2w6m.2 Direct URL to data: https://data.mendeley.com/datasets/4bt4jwjdwm/2 https://data.mendeley.com/datasets/rghjtw9p55/2 https://data.mendeley.com/datasets/wrt4dk2w6m/2
Related research article	L.R.D. Mendes, C.M. Pschenyckyj, B. Mozafari, M. Bruen, F. O'Loughlin, F. Renou-Wilson, Effluent dynamics from a degraded peatland catchment and the influence of an on-site pond—a warning sign, Journal of Hydrology (2026). [1]

## 1 Value of the Data

• This dataset provides one of the few openly available high-temporal-resolution records combining flow, temperature, and water quality measurements during 14 storm events of varying intensities in a degraded industrial peatland catchment. Such datasets are scarce and provide valuable opportunities to investigate short-term hydrochemical responses to storm events, which are often underrepresented in peatland studies. The high frequency measurements of pH, conductivity, nutrients, SUVA_254_ (carbon aromaticity), and dissolved ions support research on hydrological and biogeochemical processes in peatland effluents, supporting research on peatland management and restoration.
• The dataset can be reused to investigate how storm events influence the transport of nutrients and carbon in peatland effluents. The combined flow and water quality measurements enable analyses of the mobilization and export of dissolved organic carbon (DOC), nitrogen (TDN, NO₃-N, NH₃-N), and ions (Na, K, Ca, Mg, Cl, SO₄), as well as studies of storm-driven nutrient transport, downstream water quality, and flow-water quality interactions.
• The grab sampling dataset from the excavated treatment pond can be reused to assess water quality changes between the pond inlet and outlet. Researchers studying treatment ponds and other nature-based solutions can reuse these data to compare treatment performance with similar systems and investigate the influence of factors such as hydraulic retention time and pond volume on water quality.
• The high-frequency flow measurements are valuable for hydrological modelling and runoff analyses in drained peatland catchments. Combined with the accompanying water quality data, they can be used to calibrate and validate hydrological models, investigate flow dynamics during storm events, and improve understanding of runoff-driven nutrient transport.
• The dataset also provides a resource for studies investigating the potential effects of changing storm patterns on peatland hydrology and water quality. By combining these data with future or long-term monitoring datasets, researchers can evaluate changes in hydrological and biogeochemical responses under different climatic and management conditions.

## 2 Background

Peatland drainage associated with peat extraction alters natural hydrological processes and can increase the export of carbon, nutrients and other dissolved constituents to downstream surface waters [[2], [3], [4]]. These changes may contribute to water quality deterioration and increase the risk of ecological impacts in receiving waters [5,6]. Despite their importance, relatively few studies have combined high-frequency flow and water quality monitoring during storm events, limiting understanding of short-term hydrochemical dynamics and the performance of mitigation measures such as treatment ponds.

This data article complements a published research article that analysed hydrological and water quality dynamics in effluents from a 16.4 ha industrial cutaway peatland in Ireland, as well as the treatment performance of a pond excavated at the edge of the catchment [1]. The research article investigated how flow and temperature conditions influenced the mobilization of nutrients and carbon, particularly during storm events. While the research article presents specific findings based on these data, this data article makes the dataset openly available for reuse in broader contexts.

By providing high-resolution flow and water quality measurements, this dataset allows researchers to further explore peatland drainage impacts, storm-driven pollutant transport, and the effectiveness of treatment ponds. It also supports comparative studies on peatland systems and nature-based solutions, as well as hydrological modelling applications. By offering detailed storm event data, this dataset facilitates research on peatland effluent dynamics and supports studies aimed at improving water quality management and peatland restoration. Finally, making this dataset publicly available enhances its scientific value, enabling new research applications beyond the scope of the original study.

## 3 Data Description

**Fig. 1:**
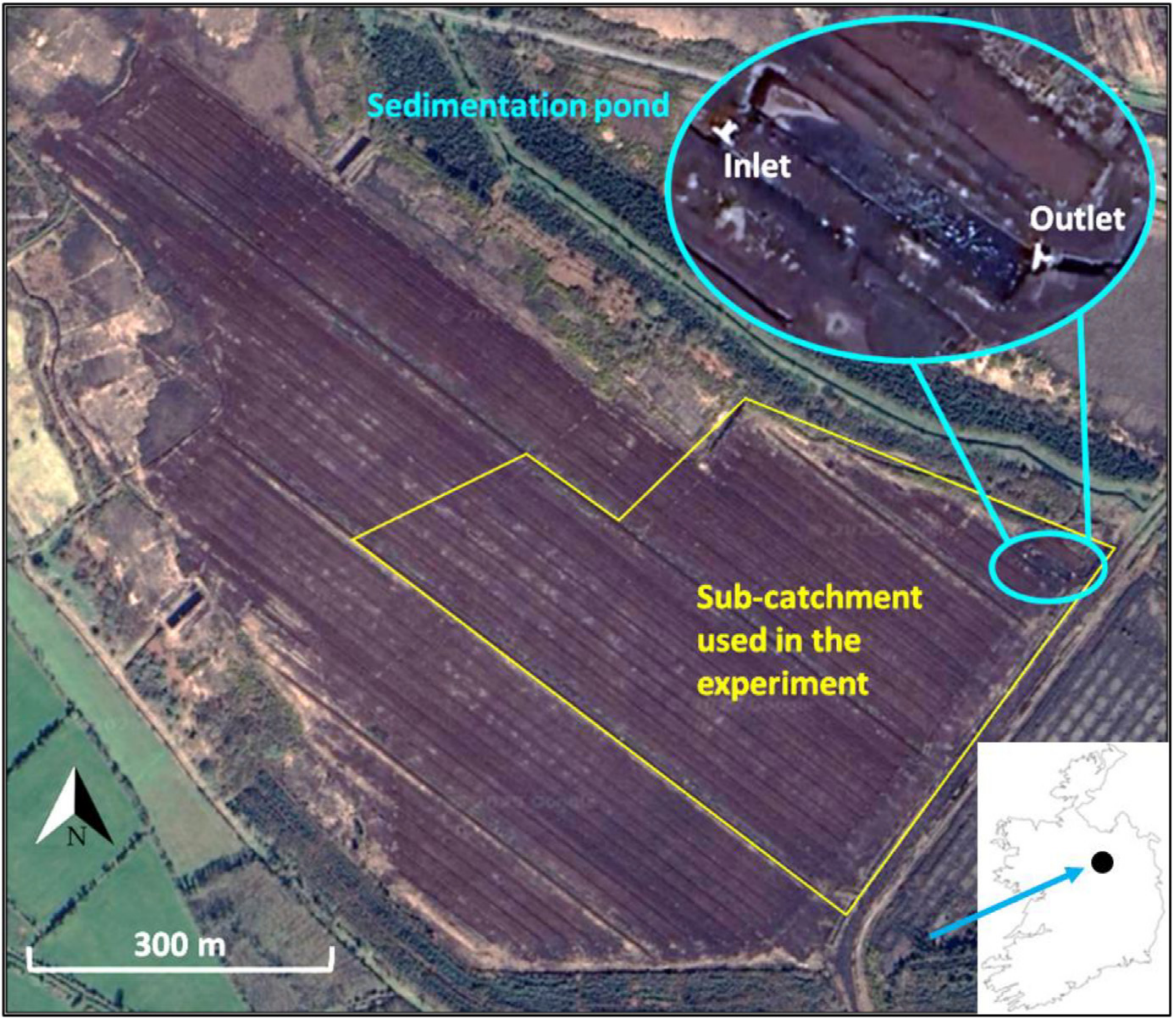
Location of the Corralanna extracted peatland catchment in Ireland, showing the monitored sub-catchment and its sedimentation pond where effluent monitoring was conducted.

The dataset consists of three structured Excel files—Storm Events [7], Flow [8], and Grab sampling [9]—designed for accessibility and reuse in further analyses. The data were collected at the outlet of an extracted peatland site in Corralanna, Ireland, where a sedimentation pond was excavated (Fig. 1). The pond covers 405 m^2^, has a volume of 959 m^3^, and receives effluents from a 16.4 ha sub-catchment. Storm Events [7] includes water quality data paired with flow and temperature measurements. Flow [8] contains high-resolution flow data recorded at 1–5-minute intervals, capturing flow dynamics such as rise, peak, and recession phases. Both datasets comprise measurements collected during 14 storm events between March 2021 and March 2023, with six events peaking below 15 L/s and eight events exceeding 30 L/s. Storm events refer to periods of heavy rainfall, causing a significant increase in flow at the sub-catchment outlet, followed by a recession phase. Grab sampling [9] comprises a separate monitoring campaign, with water quality samples collected at the inlet and outlet of the treatment pond from March to October 2021 and September 2022 to April 2023. The hydraulic retention time was estimated independently using pond volume and flow data for the period July 2022 to June 2023, yielding a median retention time of 15 days and a mean of 90 ± 551 days. The three files contain a wide range of hydrochemical variables relevant to studying effluent water quality dynamics, downstream water quality impacts, and the pond's efficiency in removing soluble components (Table 1).

**Table 1:** Overview of the variables measured in the storm events, flow, and grab sampling files available in the Mendeley data repository.

Column Name	Unit	Description
Date Time	-	Timestamp of measurement (DD-MM-YYYY HH:MM)
Sample ID	**-**	Unique identifier for each sample collected during the study
Flow	L/s	Water flow rate in litres per second
Temperature	°C	Water temperature in degrees Celsius
pH	-	Measure of water acidity/alkalinity
EC	µS/cm	Electrical conductivity, indicating ion concentration
TDC	mg/L	Total dissolved carbon concentration
DOC	mg/L	Dissolved organic carbon concentration
SUVA_254_	-	Specific UV absorbance at 254 nm (indicator of organic matter quality and aromaticity)
TDN	mg/L	Total dissolved nitrogen concentration
NO_3_-N	mg/L	Nitrate-nitrogen concentration
NH_3_-N	mg/L	Total ammonia (ammonia + ammonium)-nitrogen concentration
Na	mg/L	Sodium concentration
K	mg/L	Potassium concentration
Ca	mg/L	Calcium concentration
Mg	mg/L	Magnesium concentration
Cl	mg/L	Chloride concentration
SO_4_	mg/L	Sulphate concentration
Fe	µg/L	Iron concentration
TP	mg/L	Total phosphorus concentration
OP	mg/L	Orthophosphate concentration
BOD_5_	mg/L O_2_	Biochemical oxygen demand after 5 days
Turbidity	NTU	Measure of water clarity based on the scattering of light by suspended particles

In Storm events [7], the time interval between sampling and collection dates did not exceed seven days, with an average collection delay of three days (Table 2). This ensured that water quality samples were retrieved in a timely manner. The dataset features higher-resolution sampling during flow rises and peaks, with the first 12 samples of each event taken at 30-minute intervals. In contrast, samples collected during flow recessions were taken at either 1.5- or 2-hour intervals. The sampler used in the study had a total capacity of 24 bottles, and Sample 13 was consistently discarded because it was collected immediately after Sample 12, before the programmed time interval change to longer intervals had occurred. The number of samples collected varied across storm events, ranging from 6 to 23, and reflects differences in storm intensity profiles and durations.

**Table 2:** Sampling and collection dates for storm events, including time intervals, sampling programs used, and the number of samples collected per event.

**Storm event**	Sampling dates	Collection date	Number of days between sampling and collection	Samples program A	Samples program B	Number of samples	Notes
1	10–11/03/2021	11/03/2021	0	Samples 1–12 (15 mins)	Samples 13–16 (2 h)	16	
2	11–12/03/2021	18/03/2021	6	Samples 1–12 (30 mins)	Samples 14–24 (2 h)	23	
3	28–29/03/2021	30/03/2021	1	Samples 1–12 (30 mins)	Samples 14–24 (2 h)	23	
4	20/05/2021	27/05/2021	7	Samples 1–6 (30 mins)	-	6	
5	04–05/07/2021	05/07/2021	0	Samples 1–12 (30 mins)	Samples 14–21 (1.5 h)	20	
6	05–06/08/2021	11/08/2021	5	Samples 1–12 (30 mins)	Samples 14–24 (1.5 h)	23	
7	21/08/2021	-	-	Samples 1–6 (30 mins)	-	6	
8	26/10/2021	26/10/2021	0	Samples 1–12 (30 mins)	Samples 14–24 (1.5 h)	23	
9	30–01/12/2021	06/12/2021	5	Samples 1–12 (30 mins)	Samples 14–24 (2 h)	23	Samples frozen pre-analysis
10	5–6/02/2022	07/02/2022	1	Samples 1–12 (30 mins)	Samples 14–24 (2 h)	22	Sample 9 was missing
11	15–16/10/2022	18/10/2022	2	Samples 1–12 (30 mins)	Samples 14–24 (2 h)	22	Sample 18 was missing
12	19–20/10/2022	26/10/2022	6	Samples 1–12 (30 mins)	Samples 14–24 (2 h)	23	
13	11–12/03/2023	13/03/2023	1	Samples 1–12 (30 mins)	Samples 14–24 (2 h)	23	
14	15–16/03/2023	21/03/2023	5	Samples 1–12 (30 mins)	Samples 14–24 (2 h)	23	

## 4 Experimental Design, Materials and Methods

### 4.1 Site description

The dataset was collected in Corralanna, a raised bog and industrial cutaway peatland catchment in County Westmeath, Ireland (Fig. 1; 53°41′54.8″N 7°24′53.6″W) at an elevation of 110 m above sea level. The 16.4 ha sub-catchment was delineated using a 0.5 m resolution LiDAR-derived digital elevation model. The peat, classified as woody fen peat, had an average depth of over 5 m (after years of extraction), a pH of 4.1, and a humification degree of H6 on the von Post scale.

The artificial drainage network consisted of 42 parallel drains spaced approximately 15 m apart, lowering the water table to approximately 1 m below the peat surface. Established in the 1980s and redeveloped in 2012, this drainage system remained active during the experiment, although some ditches showed signs of natural clogging after peat harvesting ceased in 2020.

The sub-catchment drains into a sedimentation pond (49.4 m × 8.2 m × 2.4 m) designed to reduce the discharge of suspended particles. This pond was cleaned 1–2 times a year to remove excess sediment, and served as the monitoring site for assessing the effects of drainage and peat extraction on hydrology and water quality.

Vegetation reflected the site's history of intensive peat extraction, consisting mainly of bare peat colonised by pioneer species such as cotton grasses (*Eriophorum angustifolium*), purple moor grass (*Molinia caerulea*), and some heather (*Calluna vulgaris*). Naturally regenerating pine and spruce saplings are also present. *Sphagnum* mosses were largely absent at the start of the experiment but colonised some ditches over the course of the study; other mosses (*Campilopus* spp.) sparingly occurred.

### 4.2 Instrumentation and monitoring programme

**Fig. 2:**
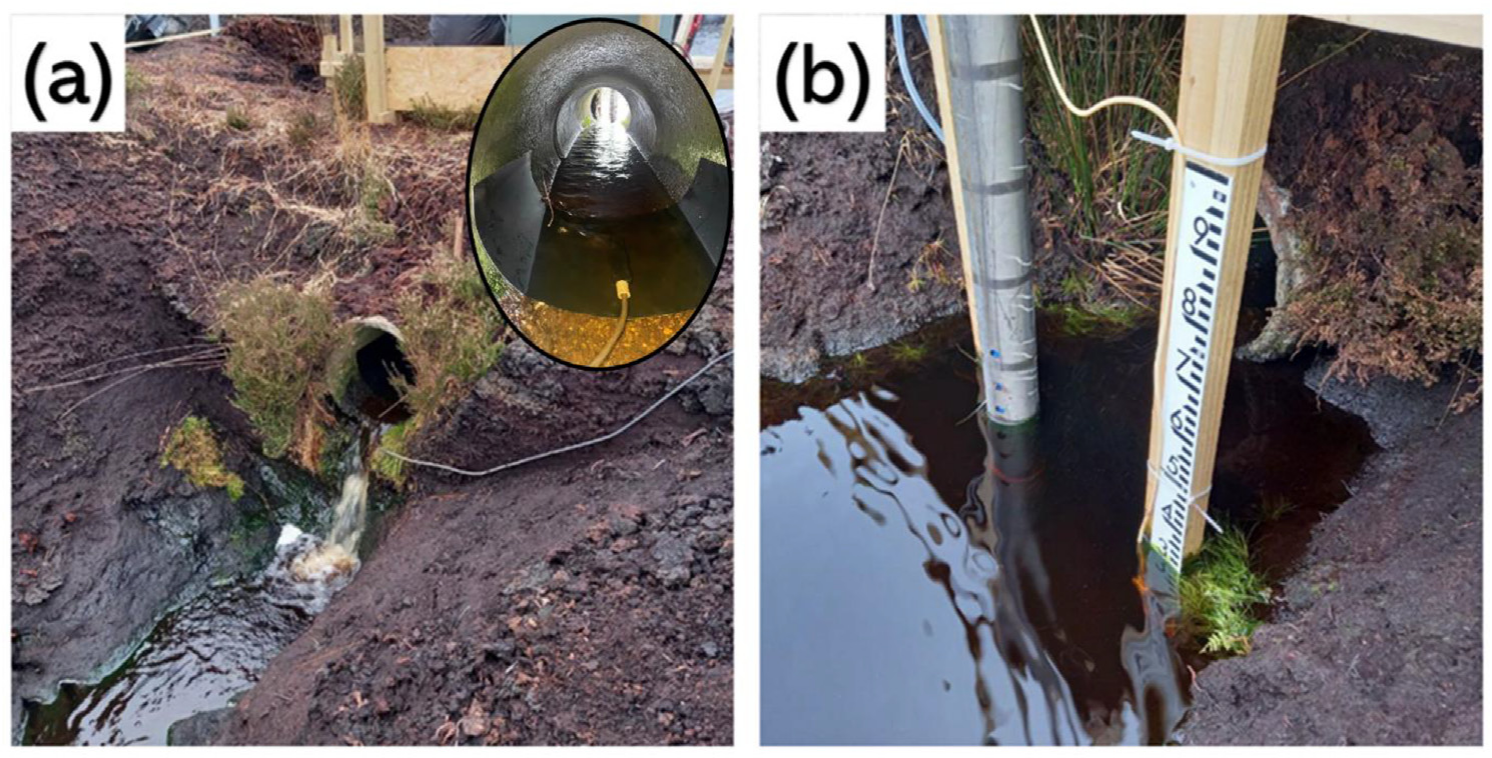
Views of the sedimentation pond outlet pipe: (a) downstream view showing the flow sensor installed inside the pipe; and (b) pond-side view showing the water level measurement and sampling location.

A monitoring platform was installed in December 2020 at the sedimentation pond’s only outlet pipe (45 cm diameter) in the sub-catchment (Fig. 2a–b). Below is a detailed description of the instruments used, including their specifications, purposes, sampling intervals, and accuracies.

#### 4.2.1 ISCO 6712 sampler

**Fig. 3:**
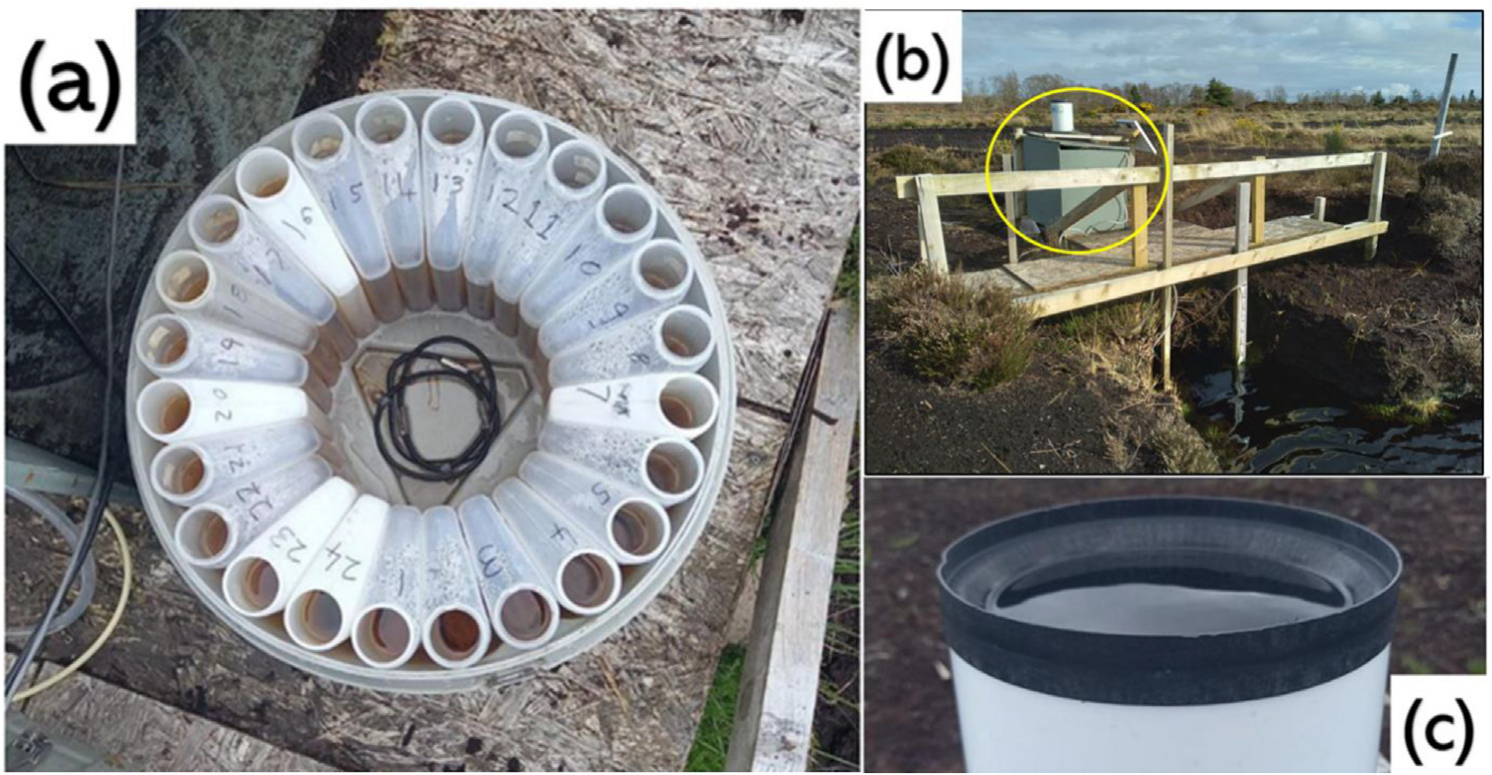
(a) Sampler container with a 24-bottle capacity. (b) Weatherproof housing enclosing the sampler and on top of the monitoring platform. (c) Example of a rain gauge blockage causing water to accumulate at the opening.

The ISCO 6712 sampler (Teledyne ISCO Inc.) was programmed to collect water samples from the pond during storm events for water quality analysis. It also functioned as the power supplier and control centre for the ISCO 730 bubbler flow module and ISCO 674 rain gauge, acting as a data collector with its internal memory. The sampler enabled wired data retrieval via a laptop using Flowlink software. It collected up to 24 bottles (1 L each) in a cyclical manner (Fig. 3a), ensuring empty bottles were always available for sample storage. When connected to the bubbler flow module, the sampler automatically triggered water collection according to predefined sampling protocols.

**Fig. 4:**
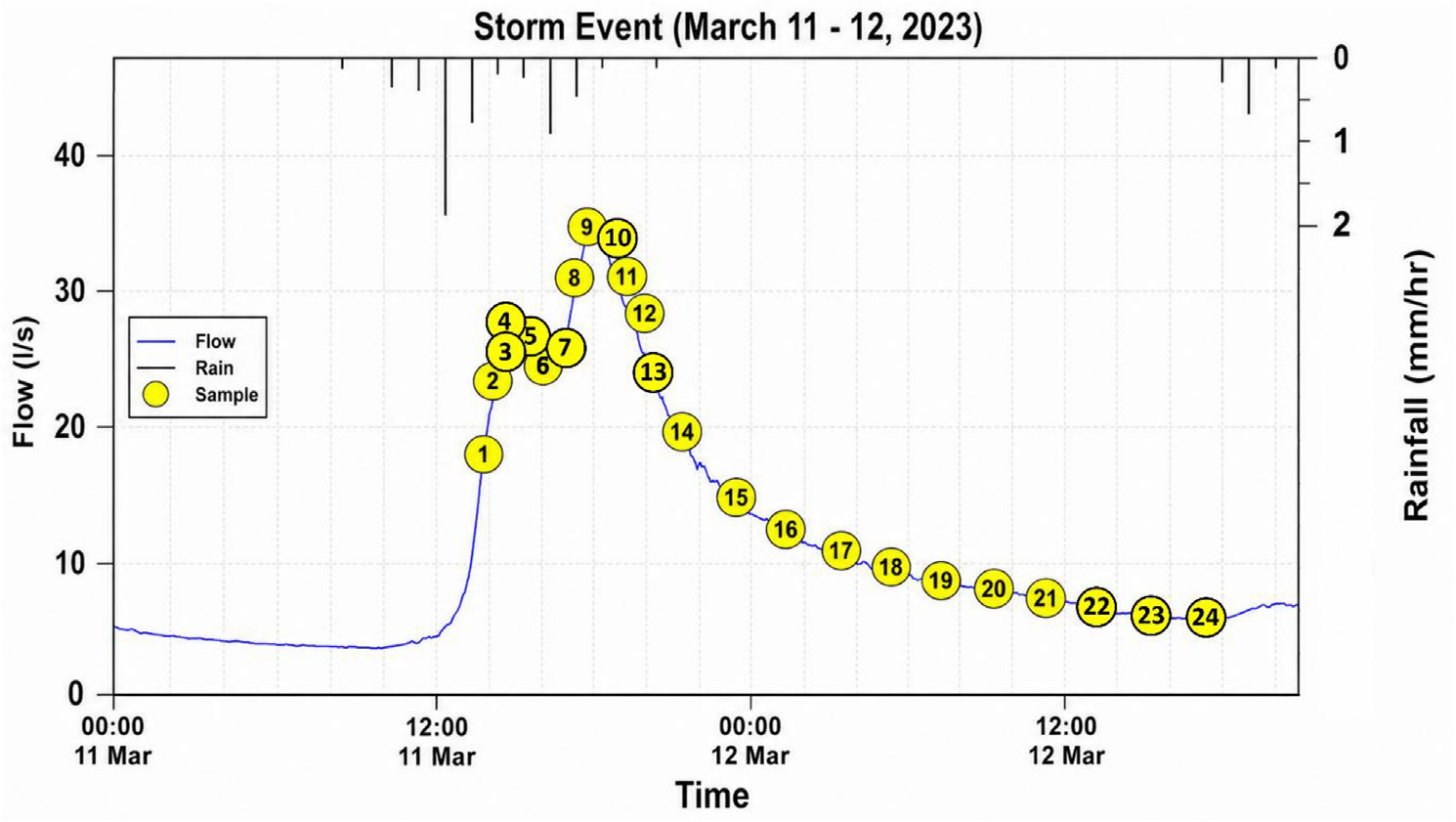
Example storm event (March 11–12, 2023) showing automated sampler programming designed to collect samples during the rising limb, peak, and recession of flow.

Storm-event sampling was initiated automatically when the pond water level exceeded predefined seasonal threshold levels that indicated rainfall-driven flow events. Once triggered, the sampler collected the first 12 samples at 30-minute intervals to capture the rising limb and peak flow, followed by the remaining samples at 1.5–2-hour intervals during the recession phase if elevated water levels persisted (Fig. 4). Trigger levels and sampling intervals were adjusted seasonally according to flow patterns, ensuring that both short-duration and longer storm events were effectively captured while maintaining correct bottle sequencing. A total of 14 storm events of varying intensities were monitored between March 2021 and March 2023.

In addition to automated storm-event sampling, grab samples were collected twice monthly from March to October 2021 at the pond outlet and monthly from September 2022 to April 2023 at both the pond inlet and outlet. Samples were transported to the laboratory on the day of collection (or, for automated samples, within seven days), stored at 2 °C, and analysed within one day of storage. Water temperature, pH and electrical conductivity were measured on site, while the remaining chemical analyses were carried out using standard accredited laboratory procedures.

The sampler was housed in a weatherproof chamber (Fig. 3b) and powered by a rechargeable 12 V 12Ah car battery, which required replacement approximately every 3–4 weeks. A 20 W solar panel, installed in July 2022, supplemented the power system by providing continuous battery charging. Due to poor network coverage at the study site, telemetric data transmission was not possible. Instead, data was downloaded manually during site visits.

#### 4.2.2 ISCO 2150 area velocity flow module

The ISCO 2150 area velocity flow module (Teledyne ISCO Inc.) combined Doppler velocity sensing with pressure-based water level measurement. It comprised a sensor head, control module, and cabling. The sensor head was securely mounted inside the pipe using a stainless-steel ring, positioned to face directly into the flow for uniform measurement (Fig. 2a). The pipe diameter was manually entered into Flowlink software to establish the cross-sectional flow area. The pressure transducer measured water level with ±0.003 m precision (range: 0.01–3.05 m). The Doppler sensor used acoustic backscattering to measure flow velocity. Discharge was calculated using the continuity equation, multiplying the cross-sectional area by the flow velocity. Discharge calculations were recorded at 5-minute intervals. The module was powered by two 6 V mercury-cadmium batteries, which lasted approximately 4 months.

**Fig. 5:**
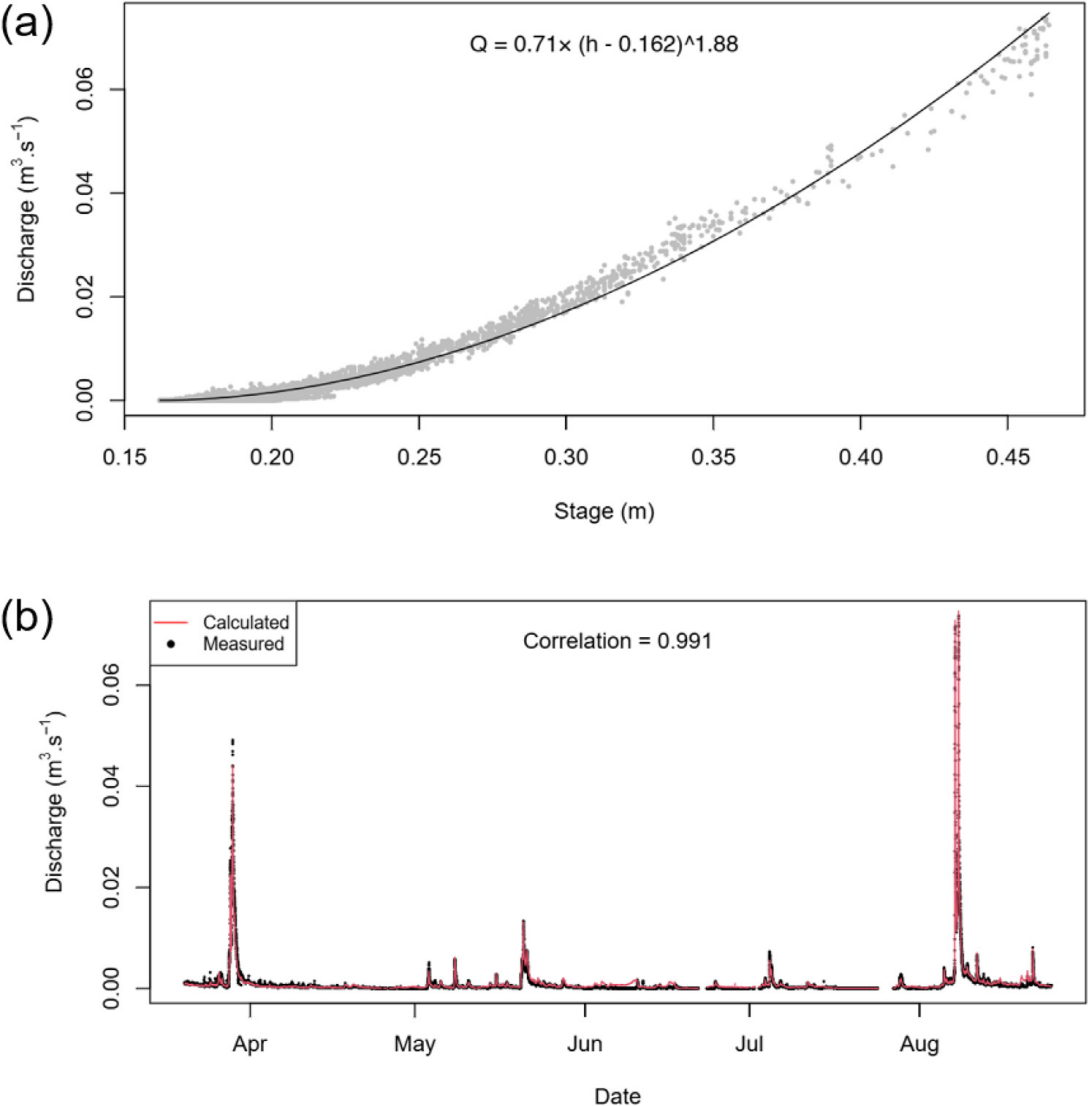
(a) Rating curve relating measured water level in the sedimentation pond (measured by the bubbler flow module) to discharge at the outlet. (b) Comparison of calculated discharge (based on the rating curve) and observed discharge (measured by the area velocity flow module) at the outlet (r = 0.99).

A stage-discharge rating curve was developed to relate water level (measured by the bubbler flow module) to discharge (from the area velocity flow module) (Fig. 5a). This enabled gap-filling in discharge data, validation of measurements, and near-continuous discharge readings. A strong positive correlation (r = 0.99) was observed between the calculated flow (based on water level) and the measured flow (from the flow meter) (Fig. 5b).

#### 4.2.3 ISCO 730 bubbler flow module

The ISCO 730 bubbler flow module (Teledyne ISCO Inc.) was used to measure water levels in the sedimentation pond by detecting changes in hydrostatic pressure. It was connected to the ISCO 6712 sampler, which recorded water levels at 5-minute intervals. The bubbler system works by maintaining air pressure in a submerged tube, where a sensor measures the pressure required to release air bubbles through an orifice at the bottom. Changes in water level cause proportional changes in hydrostatic pressure, which are converted into depth measurements. The sensor compensates for variations in temperature and air density without drift or frequent calibration, ensuring depth measurements with ±0.3 cm accuracy even in the presence of dissolved solids.

The instrument's self-cleaning mechanism prevents blockages by periodically purging the orifice with air bubbles, while the non-contact design minimizes interference from floating debris. A desiccant system removes moisture before measurement. The bubbler tube was anchored to the pond side of the outlet (Fig. 2b), ensuring the orifice remained submerged but did not touch the bottom.

#### 4.2.4 ISCO 674 rain gauge

Rainfall was measured using an ISCO 674 tipping bucket rain gauge (Teledyne ISCO Inc.), mounted atop the sampler housing at 1 m above ground level with an unobstructed sky view. The gauge was wired to the sampler for automated precipitation recording. It features a 400 cm² funnel opening that directs rainwater into a seesaw-like tipping mechanism with two small buckets. As water fills one bucket, it tips (0.25 mm per tip), emptying the water and allowing the second bucket to fill. Each tip generates a signal to the sampler, recording a precipitation event with an accuracy of ±1.5%.

The gauge was factory calibrated and field-validated by correlation against rainfall data from the Met Éireann Coolure weather station (approximately 4 km southeast at about the same elevation of 73 m) to ensure accuracy. Funnels were routinely checked for blockages, and water ponding at the opening was addressed (Fig. 3c).

## Limitations

The dataset has some inconsistencies across storm events due to missing measurements for specific parameters. For example, pH and conductivity data are absent for storm events 1, 4, and 9, while SUVA_254_ is missing for events 1, 5, 9, and 11, and partially missing in event 12. Carbon data is unavailable for events 5 and 11 and partially missing in event 12. Nitrogen and ion measurements are inconsistent across storm events, with some parameters missing in certain events, particularly between events 9 and 14. Additionally, for storm event 12, flow data is limited in magnitude and does not fully encompass all corresponding water quality measurements.

The dataset from the grab sampling campaign at the pond’s inlet and outlet is relatively limited, restricting comprehensive assessments of the pond’s treatment efficiency. It lacks consistent data across all sampling dates and water quality parameters, and several measurements, including nitrate, phosphorus, and BOD₅, often fall below the detection limit. Despite these limitations, the dataset still provides valuable insights into water treatment, as well as strong evidence of the pond’s inefficiency in treating soluble components.

## Ethics Statement

The authors have read and follow the ethical requirements for publication in Data in Brief. The current work does not involve human subjects, animal experiments, or any data collected from social media platforms.

## Credit Author Statement

**Lipe R. D. Mendes**: Conceptualisation, Data curation, Formal analysis, Investigation, Validation, Visualisation, Writing – original draft, Writing – review and editing. **Behzad Mozafari**: Data curation, Formal analysis, Investigation, Validation, Visualisation, Writing – original draft, Writing – review and editing. **Catharine M. Pschenyckyj**: Conceptualisation, Data curation, Investigation, Validation, Writing – review and editing. **Michael Bruen**: Conceptualization, Funding acquisition, Methodology, Writing – review and editing. **Fiachra O'Loughlin**: Writing – review and editing. **Florence Renou-Wilson**: Conceptualisation, Funding acquisition, Methodology, Project administration, Resources, Supervision, Writing – review and editing.

## Declaration of Competing Interest

The authors declare that they have no known competing financial interests or personal relationships that could have appeared to influence the work reported in this paper.

## Data Availability

Mendeley DataInlet and Outlet Water Quality of a Treatment Pond in an Extracted Peatland Catchment, Ireland (Original data)
Mendeley DataStorm Event Flow Data from an Industrial Cutaway Peatland, Ireland (Original data)
Mendeley DataStorm Event Water Quality in an Industrial Cutaway Peatland, Ireland (Original data)

## References

[1] Mendes L.R.D., Pschenyckyj C.M., Mozafari B., Bruen M., O’Loughlin F., Renou-Wilson F. (2026). Effluent dynamics from a degraded peatland catchment and the influence of an on-site pond—a warning sign. J. Hydrol..

[2] Holden J., Chapman P.J., Lane S.N., Brookes C., Martini I.P., Cortizas A.M., Chesworth W. (2006). Peatlands Evol. Rec. Environ. Clim. Chang..

[3] Marttila H., Karjalainen S.-M., Kuoppala M., Nieminen M.L., Ronkanen A.-K., Kløve B., Hellsten S. (2018). Elevated nutrient concentrations in headwaters affected by drained peatland. Sci. Total Environ..

[4] Nieminen M., Sarkkola S., Hellsten S., Marttila H., Piirainen S., Sallantaus T., Lepistö A. (2018). Increasing and decreasing nitrogen and phosphorus trends in runoff from drained peatland forests—is there a legacy effect of drainage or not?. Water, Air, Soil. Pollut..

[5] Evans C.D., Renou-Wilson F., Strack M. (2016). The role of waterborne carbon in the greenhouse gas balance of drained and re-wetted peatlands. Aquat. Sci..

[6] Nieminen M., Sarkkola S., Sallantaus T., Hasselquist E.M., Laudon H. (2021). Peatland drainage - a missing link behind increasing TOC concentrations in waters from high latitude forest catchments?. Sci. Total Environ..

[7] Mendes L.R.D., Pschenyckyj C., Mozafari B., Bruen M., O’Loughlin F., Renou-Wilson F. (2025). Storm event water quality in an industrial cutaway peatland, Ireland. Mendeley Data..

[8] Mendes L.R.D., Pschenyckyj C., Mozafari B., Bruen M., O’Loughlin F., Renou-Wilson F. (2025). Storm event flow data from an industrial cutaway peatland, Ireland. Mendeley Data..

[9] Mendes L.R.D., Pschenyckyj C., Mozafari B., Bruen M., O’Loughlin F., Renou-Wilson F. (2025). Inlet and outlet water quality of a treatment pond in an extracted peatland catchment, Ireland. Mendeley Data..

